# Development of Biomimetic Edible Scaffolds for Cultured Meat Based on the Traditional Freeze-Drying Method for *Ito-Kanten* (Japanese Freeze-Dried Agar)

**DOI:** 10.3390/gels11040299

**Published:** 2025-04-18

**Authors:** Ping Xia, Hiroki Miyajima, Satoshi Fujita

**Affiliations:** 1 Department of Frontier Fiber Technology and Sciences, University of Fukui, Fukui 910-8507, Japanh.miyajm@u-fukui.ac.jp (H.M.); 2 Life Science Innovation Center, University of Fukui, Fukui 910-8507, Japan

**Keywords:** soy protein isolate, carrageenan, sodium alginate, cryogel, anisotropy, cultured meat

## Abstract

In this study, we aimed to develop soy protein-derived edible porous hydrogel scaffolds for cultured meat based on mechanical anisotropy to mimic the physical and biochemical properties of muscle tissues. Based on the traditional Japanese *Ito-Kanten* (thread agar) freeze–thaw process, we used liquid nitrogen directional freezing combined with ion crosslinking to fabricate an aligned scaffold composed of soy protein isolate (SPI), carrageenan (CA), and sodium alginate (SA). SPI, CA, and SA were dissolved in water, heated, mixed, and subjected to directional freezing in liquid nitrogen. The frozen gel was immersed in Ca^2+^ and K^+^ solutions for low-temperature crosslinking, followed by a second freezing step and lyophilization to create the SPI/CA/SA cryogel scaffold with anisotropic pore structure. Furthermore, C2C12 myoblasts were seeded onto the scaffold. After 14 d of dynamic culture, the cells exhibited significant differentiation along the aligned structure of the scaffold. Overall, our developed anisotropic scaffold provided a biocompatible environment to promote directed cell differentiation, showing potential for cultured meat production and serving as a sustainable protein source.

## 1. Introduction

Sustainable food production methods are urgently needed to meet the growing protein demand worldwide. Cultured meat, produced via in vitro cell culture, offers a scalable alternative that replicates the taste, texture, and nutritional profile of conventional meat, with reduced environmental impact [1,2]. Plant- and cell-based alternatives are used to overcome the limitations of traditional animal agriculture, with plant-derived proteins being safer, healthier, and more sustainable than conventional proteins [3,4,5]. Advances in processing technologies have further improved the resemblance of cultured meat to conventional meat [6,7].

Scaffolds are essential for cultured meat production, providing structural support that mimics the in vivo microenvironment and promotes cell adhesion, proliferation, and tissue development [8,9]. Porous materials and hydrogels are particularly promising owing to their low cost, simple fabrication process, and ability to replicate the extracellular matrix, creating a physiologically relevant culture environment [10]. Plant-based scaffolds are ideal biomaterials for cultured meat production.

Soy protein isolate (SPI), a high-quality plant protein with extracellular matrix-like biochemical properties, is widely used in the food industry [11]. However, SPI hydrogels exhibit poor mechanical strength and water resistance, limiting their use as scaffolds. To address this, SPI is often combined with natural polymers to improve its mechanical properties and support cell adhesion and proliferation [12,13]. We previously developed an SPI-based hydrogel incorporating edible polysaccharides, carrageenan (CA), and sodium alginate (SA), and demonstrated its potential as a fully plant-derived edible scaffold for cell cultures.

CA, a sulfated polysaccharide derived from red seaweed, forms thermally reversible hydrogels via a three-dimensional network of double helices. Its gelation depends on the interactions with monovalent and divalent cations, forming a rigid structure; CA-based hydrogels are widely used in various industries, including the food industry [14,15,16]. SA, a polysaccharide derived from brown algae, forms durable “egg-box” structures in the presence of divalent cations, such as Ca^2+^, and is commonly used in the food and medical industries [17,18].

Despite their advantages in terms of physical properties and edibility, hydrogels lack anisotropic properties, which are crucial for mimicking the fibrous structure of natural meat. Directed cell growth is essential for the formation of functional muscle tissues. The alignment of scaffold pores or fibers creates an anisotropic structure that improves cell attachment, differentiation, and structural integrity. This also contributes to a muscle-like texture and accelerates tissue formation [19,20]. Anisotropic hydrogel scaffolds are essential for tissue engineering and cultured meat production, providing structural and mechanical reinforcement by guiding cell alignment and replicating the biological tissue organization. However, forming hydrogel scaffolds with precise molecular orientation and structural anisotropy remains challenging [21].

*Kanten* (from Japanese), a polysaccharide derived from red algae such as *Gelidium* and *Gracilaria*, consists of neutral agarose and acidic agaropectin, showing varying physicochemical properties depending on its extraction and purification methods. *Ito-Kanten* (thread agar) is traditionally produced in Japan via freeze–thaw cycling, with the raw seaweed (*Tengusa*) undergoing repeated freezing and thawing over two nights under outdoor winter conditions [22,23,24]. This process induces molecular orientation, forming an anisotropic network that enhances the mechanical strength and water-holding capacity of the hydrogel. The anisotropic structure in *Kanten* is primarily formed via ice crystal templating, where directional ice growth aligns the agar molecules during freezing, and controlled thawing further refines this orientation. Subsequent freeze-drying removes the ice crystals, leaving a parallel-aligned porous structure that reinforces the anisotropic properties [25,26,27]. Cold processing techniques further optimize these properties and improve the gelation behavior and mechanical anisotropy.

**Scheme 1:**
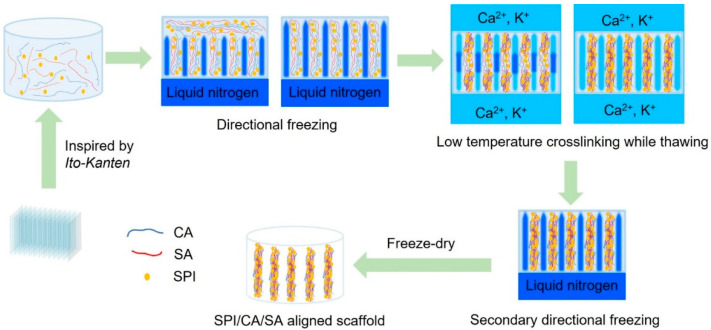
Schematic illustration of the fabrication of the aligned scaffold.

Building on the freeze–thaw process, we established SPI/CA/SA scaffolds with anisotropic porous structures by combining CA, known for its thermally reversible behavior; SA, which rapidly crosslinks with metal ions; and SPI, which promotes excellent cell adhesion. Two-time directional freezing and ion crosslinking were used to fabricate the scaffolds. During directional freezing, ice crystals grew along a temperature gradient from a cold source to a heat source, resulting in parallel-aligned ice structures. After thawing and freeze-drying, a porous scaffold with a directional pore structure was obtained (Scheme 1).

Applying the above-mentioned approach to cultured meat, we aimed to develop scaffolds supporting cell adhesion and proliferation and promoting the structured growth of muscle cells. By replicating the fibrous structure of muscle tissues, this technique enabled the scalable production of structured cultured meat with enhanced sensory and nutritional qualities. Ultimately, cultured meat is a promising alternative to conventional meat, contributing to a more sustainable food system.

## 2. Results

### 2.1. Morphology of the SPI/CA/SA Scaffold

In this study, anisotropic gels formed by combining SPI and the edible polysaccharides SA and carrageenan (CA) were investigated. Based on our previous report, we examined various compositions (Tables S1 and S2; Figures S1–S4) and determined the optimal formulations (Table 1).

**Figure 1:**
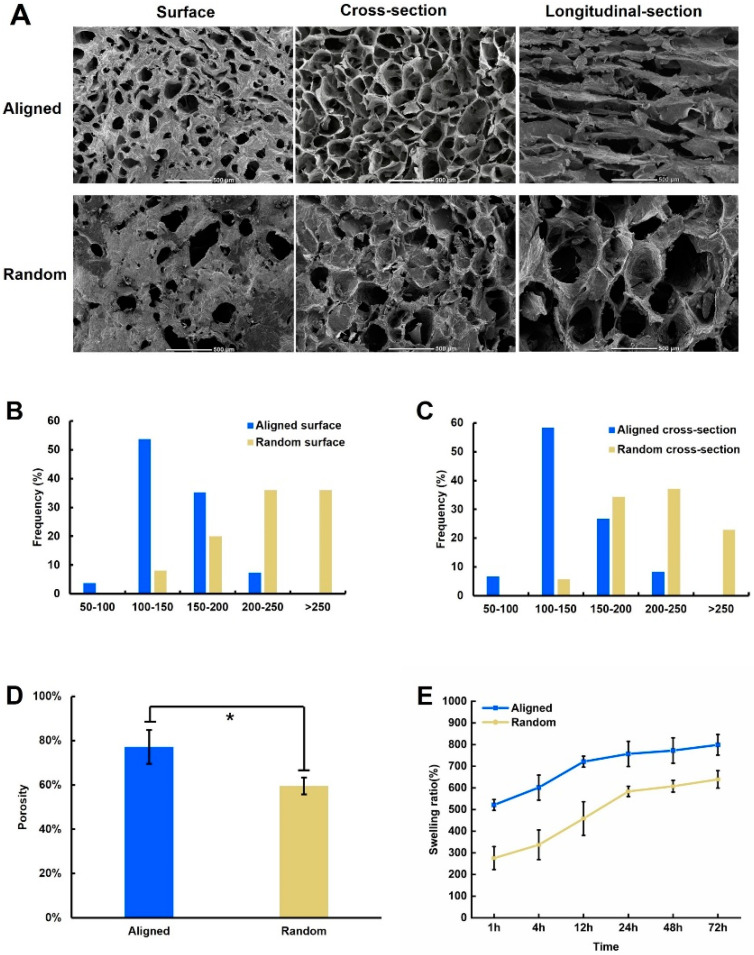
Structural and physical properties of soy protein isolate (SPI)/carrageenan (CA)/sodium alginate (SA) aligned and random cryogel scaffolds: (**A**) Scanning electron microscopy (SEM) images showing the surface, cross-section, and longitudinal section morphologies of the aligned and random scaffolds. (**B**) Pore size distribution on the scaffold surface. (**C**) Pore size distribution in the cross-section. (**D**) Porosity percentage determined from the SEM images. (**E**) Swelling ratio over time. Scale bars = 500 µm. Data are represented as the mean ± standard deviation (SD; *n* = 3; * *p* < 0.05).

SPI/CA/SA aligned and random scaffolds were obtained via the directional and conventional freeze-drying methods, respectively. SEM was used to analyze the scaffolds. As shown in Figure 1A, pores were formed on the top surface of both cylindrical scaffolds. In the aligned scaffolds, this surface corresponded to the side farthest from the cold source during directional freezing and exhibited a more uniform pore distribution.

Typically, pores larger than 100 μm in size allow cell migration and spreading within the scaffold. Pore size on the surface and cross-sections was mainly distributed within 100–150 μm in the aligned scaffolds and 200–250 μm in the random scaffolds. Both scaffolds exhibited sufficient pore sizes for cell infiltration (Figure 1B,C). The porosities of the aligned and random scaffolds were approximately 77 and 59%, respectively (Figure 1D).

### 2.2. Swelling Ratio

Owing to their porous structures, both scaffolds exhibited high swelling ratios in the early stages. After 24 h, swelling equilibrium was reached, with the aligned scaffolds achieving a swelling ratio of over 700%, whereas the random scaffolds reached a swelling ratio of over 580% (Figure 1E). The highly ordered pore structure of the aligned scaffolds facilitated rapid water infiltration and diffusion along the pores. In contrast, random scaffolds exhibited lower water absorption capacity due to their disordered pore structure, resulting in a longer water diffusion path.

### 2.3. Anisotropy

**Figure 2:**
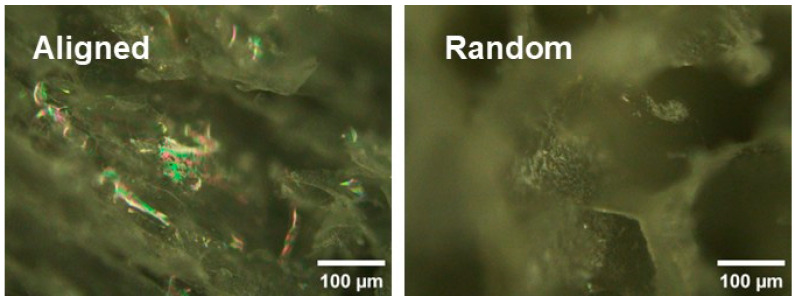
Birefringence observation of the aligned and random cryogel scaffolds via polarized microscopy. Scale bars = 100 μm.

To investigate anisotropy, both scaffolds were sliced lengthwise, and birefringence was examined via polarized light microscopy. As shown in Figure 2, when the main orientation of the aligned scaffolds was set at 45° relative to the polarization direction, strong birefringence was observed, indicating that the parallel tubular pore structure of the aligned scaffolds was anisotropic. In contrast, random scaffolds did not exhibit significant birefringence, indicating that their disordered pore structure lacked anisotropy.

### 2.4. Mechanical Properties

**Figure 3:**
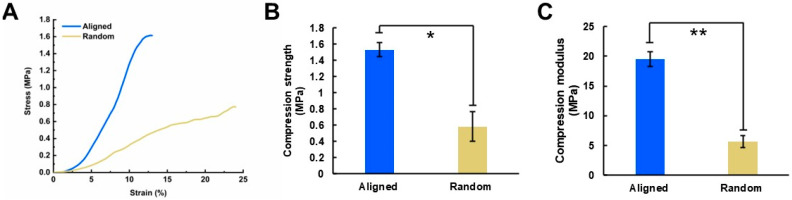
Mechanical properties of the aligned and random cryogel scaffolds determined via compression tests: (**A**) Compression stress–strain curves, (**B**) compression strengths, and (**C**) compression moduli of both scaffolds (*n* = 3; * *p* < 0.05 and ** *p* < 0.01).

As shown in Figure 3A–C, the aligned scaffolds exhibited superior mechanical properties, including greater compressive strength and higher Young’s modulus, than the random scaffolds. This improvement was attributed to the presence of directional channels in the aligned scaffolds, which enhanced the structural stability by providing a more effective supporting structure. Figure S5 shows the mechanical anisotropy. In contrast, random scaffolds, characterized by large and irregularly distributed pores, exhibited reduced stability, which negatively affected their compressive strength [28].

**Figure 4:**
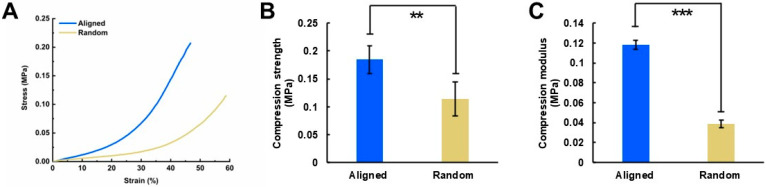
Mechanical properties of the aligned and random scaffolds in the hydrogel state determined via compression tests: (**A**) Compression stress–strain curves, (**B**) compression strengths, and (**C**) compression moduli of both scaffolds (*n* = 3; ** *p* < 0.01 and *** *p* < 0.001).

Figure 4A–C shows the compression curves and moduli, highlighting the mechanical behaviors of the scaffolds in their hydrogel state. In this state, both scaffolds showed a significant reduction in compressive strength and modulus, indicating a soft structure. However, their strain capacity and elasticity were notably enhanced in the hydrogel state compared to those in the cryogel state. Specifically, the aligned scaffolds exhibited superior compressive strength and modulus, reinforcing their enhanced robustness under compression.

### 2.5. Fourier-Transform Infrared (FTIR) Spectroscopy

**Figure 5:**
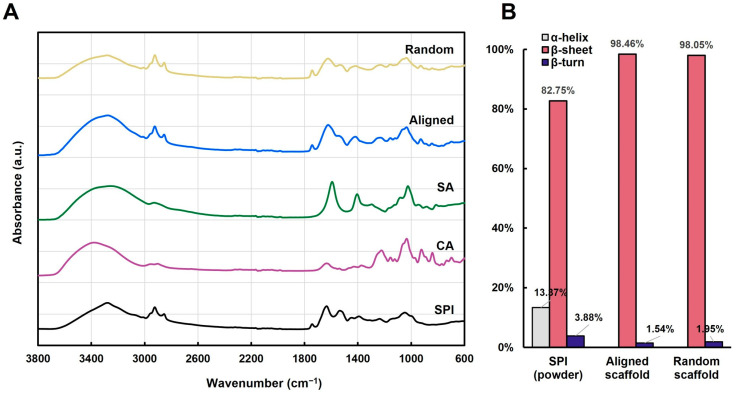
(**A**) Fourier-transform infrared (FTIR) spectra of the aligned and random cryogels, with spectra of the individual components (SPI, CA, and SA) shown for comparison. (**B**) Proportions of protein secondary structures determined via the deconvolution of the amide I peak in the FTIR spectra.

The aligned and random scaffold structures were characterized via FTIR spectroscopy, and the spectra of the raw materials (SPI, CA, and SA) were also assessed for comparison (Figure 5A). The characteristic peaks observed in the spectra of both scaffolds corresponded to those of SPI, CA, and SA, with no new peaks detected. This confirmed that the hydrogel formation did not involve covalent molecular interactions but was stabilized primarily via electrostatic interactions and hydrogen bonding, consistent with a previous report [29].

Both scaffolds exhibited a shift to lower wavenumbers within the broad peak range of 3500–3200 cm^−1^, indicating the formation of strong hydrogen bonds between the N-H groups of SPI and hydroxyl groups of SA and CA. These interactions enhanced the hydrogel stability and promoted protein rearrangement and aggregation [30]. Upon a comparison of peak positions, the aligned scaffolds showed a peak shift to 3270 cm^−1^, whereas the random scaffolds showed a peak shift to 3275 cm^−1^. The greater shift in the aligned scaffolds indicated a higher degree of hydrogen bond formation, possibly due to directional freezing induced by liquid nitrogen. This process facilitated the formation of aligned ice crystals and promoted the ordered rearrangement of polymer molecules within the gel network. In contrast, the random scaffolds lacked this ice-templating effect, thereby showing a more disordered molecular arrangement and lower degree of hydrogen bond formation than the aligned scaffolds.

Further shifts were observed in the asymmetric and symmetric stretching peaks of CO–O groups. In the aligned scaffolds, these peaks shifted from 1593 and 1403 cm^−1^ to 1624 and 1419 cm^−1^, respectively. In the random scaffolds, they shifted to 1624 and 1422 cm^−1^, respectively. These shifts suggest that SA crosslinks with calcium ions to form ionic bonds during gel formation. Additionally, the CA sulfate group peak shifted from 1224 to 1238 cm^−1^ in the aligned scaffolds and 1233 cm^−1^ in the random scaffolds. This shift indicates electrostatic interactions between sulfate groups and K^+^ ions, with the greater shift in the aligned scaffolds suggesting stronger crosslinking. The broad peak near 1037 cm^−1^ in both scaffolds was due to the overlapping interactions of the C–O groups in SPI, CA, and SA.

### 2.6. Secondary Structure Analysis

Amide I band (1600–1700 cm^−1^) is a highly sensitive spectral region used to analyze secondary protein structures [31]. The ratio of absorbance areas within this range is used to quantify the α-helix, β-sheet, β-turn, and random coil structures [8,32,33]. Here, the β-sheet content was determined at 1610–1640 and 1660–1680 cm^−1^, whereas the α-helix, β-turn, and random coil contents were analyzed at 1650–1600, 1680–1695, and 1660–1650 cm^−1^, respectively.

In its powdered state, SPI consisted of 82.8% β-sheet and 13.4% α-helix. In contrast, aligned scaffolds exhibited a β-sheet content of 98.5%, whereas the random scaffolds exhibited a slightly lower β-sheet content of 98.1% (Figure 5B). This significant increase in β-sheet content in both scaffolds was due to the freeze–thaw process that promoted protein crystallization and caused substantial secondary structure changes during hydrogel formation.

### 2.7. Cell Morphology

**Figure 6:**
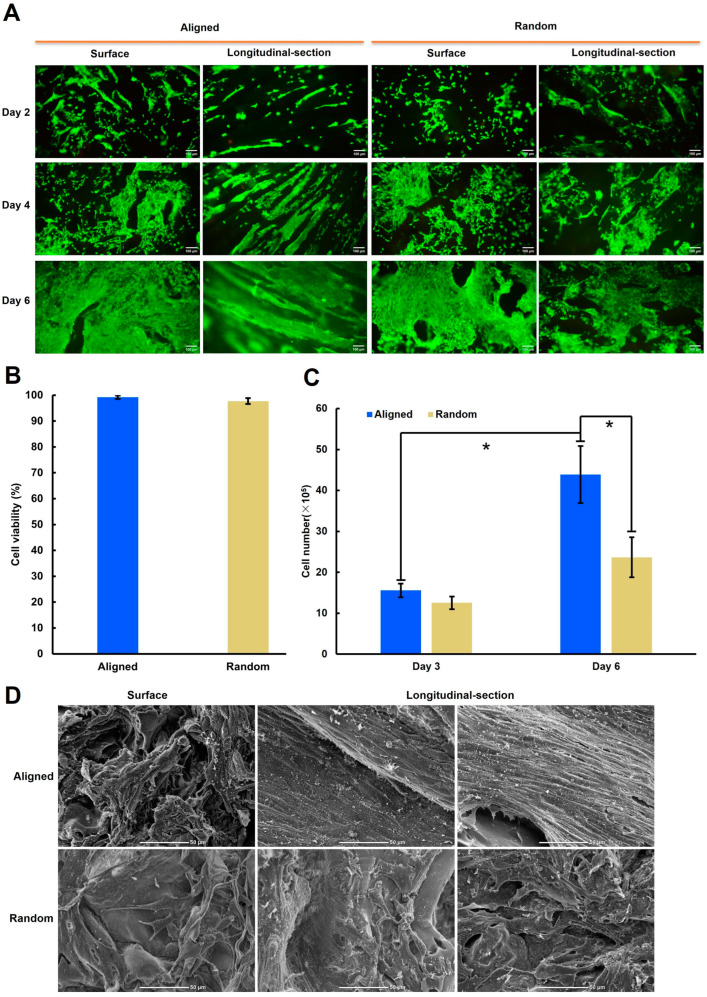
(**A**) Cellular morphologies of the C2C12 cells on both scaffolds. Fluorescence microscopy images show cell adhesion and proliferation on the cryogel surface and longitudinal section on days 2, 4, and 6. Scale bars = 100 µm. (**B**) Cell viability at day 6. (**C**) Quantitative analysis of cell proliferation on both scaffolds over time. The graph shows the cell counts on days 3 and 6 post-seeding, starting with an initial seeding density of 5 × 10^5^ cells. Data are represented as the mean ± SD (*n* = 3; * *p* < 0.05). (**D**) SEM images of the surfaces and longitudinal sections of both scaffolds on day 7 showing the cell morphology and distribution. Surface scale bar = 50 µm.

C2C12 myoblasts were cultured on both the aligned and random SPI/CA/SA scaffolds at a seeding density of 5 × 10^5^ cells/sample. From day 3, the culture containers were shaken on an orbital shaker. Cell viability was evaluated via live/dead staining on days 2, 4, and 6 to determine the scaffold compatibility (Figure 6A). Staining confirmed the absence of cytotoxic effects in both scaffolds. On day 6, cell viability exceeded 99% on the aligned scaffolds and 98% on the random scaffolds, indicating the non-cytotoxicity of both scaffolds (Figure 6B). On day 2, only a small number of cells attached to the scaffold surfaces. On day 4, the cells proliferated rapidly on both scaffolds and transitioned from a round to an elongated morphology. The cells were distributed around the scaffold pores, facilitating their migration to the interior. On day 6, the C2C12 cells covered both scaffolds and exhibited a fibrous morphology. The cells grew along the parallel tubular pores in the longitudinal cross-sections of the aligned scaffolds, but they exhibited multidirectional growth in accordance with the disordered pore distribution on the random scaffolds.

An SEM analysis on day 7 (Figure 6D) revealed substantial cell infiltration in both scaffolds. Many cells attached to the scaffold surfaces migrated inward along the scaffold pores. In the longitudinal sections, the cells penetrated and grew within both scaffolds. The cells exhibited parallel fibrous growth along the tubular pores on the aligned scaffolds, whereas the cell orientation was disorganized on the random scaffolds.

### 2.8. Cell Growth

Initially, 5 × 10^5^ C2C12 cells were seeded onto both scaffolds. After three days of culture, cell proliferation in the aligned scaffolds formed (1.56 ± 0.17) × 10^6^ cells, which further increased to (4.39 ± 0.70) × 10^6^ cells on day 6 (Figure 6C). In contrast, the cells in the random scaffolds showed slightly lower proliferation on day 3, forming (1.25 ± 0.16) × 10^6^ cells, which significantly decreased to (2.37 ± 0.49) × 10^6^ cells by day 6. On days 3–6, the proliferation rate was markedly higher on the aligned scaffolds than on the random scaffolds.

### 2.9. Cell Differentiation

**Figure 7:**
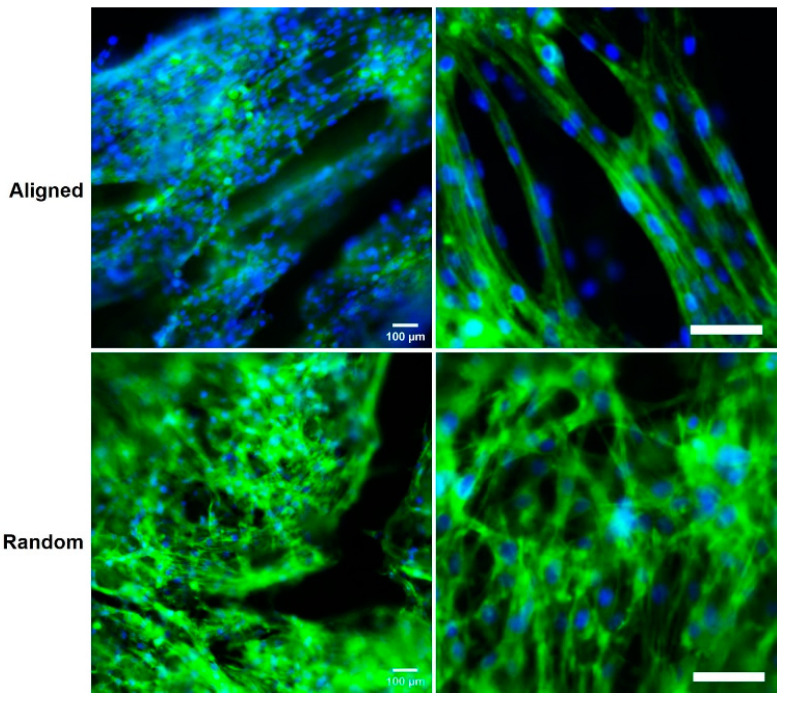
C2C12 cell differentiation in the aligned and random scaffolds. C2C12 cells on the scaffolds with the aligned and random porous structures were stained with phalloidin (green) and Hoechst (blue) after 14 d of culture. Scale bars = 100 µm.

To investigate the effects of the aligned and disordered porous structures on C2C12 differentiation, immunofluorescence staining was performed on both scaffolds on day 14. As shown in Figure 7, the C2C12 cells exhibited a non-random linear distribution on the aligned scaffolds, whereas disorganized microfilaments were observed on the random scaffolds. In contrast, cell differentiation occurred in multiple directions, and the resulting tissue structure was relatively random, making it difficult to form functional and directionally consistent tissues.

## 3. Discussion

### 3.1. Use of Scaffolds for Cultured Meat Production and Design Requirements

The current food production system, particularly the livestock sector, cannot sustainably meet the rising global demand for meat. Cultured meat is a key alternative to address future global food security challenges by enabling meat production using resource-efficient methods [1,2]. However, the cultured meat industry depends heavily on scaffolds, as appropriate scaffolds facilitate cell adhesion, proliferation, and tissue development. Scaffolds of collagen and gelatin are commonly used in this industry; however, currently, the industry is facing a shortage of edible non-animal-derived materials suitable for cultured meat production [3,4,5]. Aligned scaffolds offer distinct advantages for cell differentiation by guiding the cells for specific attachment, proliferation, differentiation, and maturation, resulting in a structure that closely mimics the native 3D microenvironment of meat tissues. Highly aligned materials are particularly desirable as they induce the formation of oriented muscle fibers, producing cultured meat with a texture similar to that of conventional meat [34].

### 3.2. Materials and Fabrication Methods of Scaffolds

Previously, we combined low-cost SPI with natural polysaccharides CA and SA to fabricate porous, edible, and aligned scaffolds [35]. Repeated heating–cooling cycles promote a more evenly distributed double-helix structure of CA, enhancing the mechanical strength and cell adhesion [35]. A directional freezing technique and low-temperature salt ion crosslinking method based on the manufacturing process of *Ito-Kanten* (thread agar) were used in this study. To optimize scaffold fabrication, a combination of directional freezing, low-temperature ionic crosslinking, and secondary directional freezing was applied following the principles of *Ito-Kanten* processing [22,25,26,27].

Directional freezing played a critical role in establishing the aligned porous structures. The rapid formation of ice crystals in the hydrogel slurry aligns them along a temperature gradient, creating a frozen hydrogel with a parallel pore structure [36]. Low-temperature ionic crosslinking was performed to further enhance their mechanical properties. The reaction between Ca^2+^ and SA formed a robust outer hydrogel layer, generating an eggshell-like structure (Figure 1A).

During controlled melting at 4 °C, K^+^ and Ca^2+^ gradually diffused into the hydrogels, inducing localized structural deconstruction while preserving the orientation. CA provided thermal stability via thermoreversibility and K^+^-mediated double-helix stabilization, whereas Ca^2+^ crosslinked both SA and CA, forming a dual-network structure. This process is similar to a previously reported method controlling protein crystallinity by regulating the water molecule content [37].

The removal of ice crystals, followed by ionic crosslinking, further refined and stabilized the aligned porous structure and improved the structural integrity (Figure 1). Secondary directional freezing after ion crosslinking ensured that the ice crystals grew along the same path, correcting incomplete internal alignment and promoting a more uniform distribution of directional ice crystals. Finally, freeze-drying removed the internal ice crystals, yielding anisotropic SPI/CA/SA scaffolds with dense pore walls and aligned pore channels.

### 3.3. Structural Analysis and Mechanical Properties

An FTIR analysis revealed that during gelation, both scaffolds exhibited a shift toward lower wavenumbers in the broad 3500–3200 cm^−1^ region (Figure 5A), indicating extensive hydrogen bonding among SPI, CA, and SA, contributing to the formation of a network structure. This network not only reinforced the mechanical properties and facilitated uniform pore formation but also promoted protein rearrangement and aggregation. Furthermore, both scaffolds exhibited higher β-sheet formation than the pure SPI powder, suggesting the involvement of intramolecular hydrogen bonding [37,38].

Directionally frozen scaffolds contained higher β-sheet contents than the randomly frozen scaffolds (Figure 5B). This enhancement was due to the unidirectional growth of ice crystals, which promoted additional hydrogen bonding, leading to a more stable and mechanically robust structure. Additionally, low-temperature ionic crosslinking during melting resulted in the gradual accumulation of hydrogen bonds and molecular interactions, further strengthening the polymer chains along the primary alignment direction. This significantly improved the longitudinal mechanical properties, resulting in mechanical anisotropy.

Repeated directional freezing formed anisotropically aligned scaffolds with parallel channels, effectively dispersing and transferring the external pressure, reducing the local stress concentration, and enhancing the mechanical strength (Figure 3 and Figure 4). Freeze-drying removed water, stabilizing the directional network and reinforcing the β-sheet structure, yielding uniform and dense scaffolds. Additionally, low-temperature ionic crosslinking was performed after the initial directional freezing step. At 4 °C, the slow melting of internal ice crystals within the aligned scaffold reduced the penetration of Ca^2+^ and K^+^ ions, facilitating gradual crosslinking. This controlled reaction promoted uniform crosslinking and solidified the internal pore alignment, further enhancing the scaffold stability. Furthermore, the interaction between SA and Ca^2+^ ions at the surface formed an eggshell-like structure, enhancing the compressive strength of the aligned scaffold.

The random scaffolds with disordered internal pores exhibited a compromised load distribution, resulting in structural weakness under pressure and reduced compressive strength. Despite these differences, both scaffolds exhibited compressive strengths > 0.4 MPa, with compression moduli at 1–5% strain surpassing those in bovine muscles under comparable conditions at room temperature [39].

### 3.4. Effects of Scaffold Porosity on Cell Proliferation

Porosity is a key factor promoting cell proliferation because it provides space for cell migration and organization. High porosity increases the available surface area for cell adhesion, facilitating cellular invasion and firm attachment, thereby establishing a stable foundation for growth. Generally, pore sizes > 100 μm optimize the nutrient and oxygen transport while efficiently removing any metabolic waste, fostering a healthy cellular environment [40].

The size of the cryogel network critically affects muscle cell adhesion and growth. Microscale pore structures, particularly aligned channels, mimic the native extracellular matrix, increase the surface area for cell attachment, and enhance adhesion. In aligned scaffolds, these channels also promote capillary-driven medium transport, improving cell migration and nutrient delivery [41,42]. Our results confirmed that aligned microscale networks support improved muscle cell adhesion and spreading, consistent with the previous findings.

In the aligned scaffolds, tubular channels were directionally arranged, creating continuous and uniform pathways maximizing the internal space and providing abundant adhesion sites for the C2C12 cells. This structured design promoted cell distribution and proliferation. Additionally, interconnected pores enhanced fluid transport within the scaffold, ensuring balanced nutrient and oxygen distribution and preventing localized hypoxia and nutrient depletion. The predominant pore size of 100–150 μm further facilitated cell migration and nutrient exchange.

The random scaffolds exhibited non-uniform pore distribution, leading to enclosed spaces and dead zones reducing the overall porosity and disrupting the structural continuity. This irregular structure impeded cell migration and nutrient transport, limiting cell proliferation. Although high porosity compromises the mechanical properties, the directional pathways in the aligned scaffolds effectively transfer and disperse the mechanical stress, allowing both high porosity and sufficient mechanical strength [43,44].

Another crucial factor for a scaffold is its swelling rate, which influences nutrient delivery, cellular microenvironment, and mechanical properties [45]. Although the aligned scaffold has a relatively small pore size, its high porosity and parallel channel structure induce a capillary effect that facilitates water infiltration along the channels, resulting in a higher swelling ratio. In contrast, the random scaffolds exhibit larger pore sizes, but their lower porosity is caused by a smaller number of pores and poor interconnectivity which limits water adsorption and diffusion, leading to a lower swelling ratio. In the aligned scaffolds, the uniform arrangement of the tubular channels facilitated smooth water and nutrient flow along the channel direction, significantly enhancing permeability. This structure also increased the interaction between hydrophilic groups and water molecules, improving the nutrient and oxygen transport by the scaffolds [46]. After 72 h, both scaffolds reached expansion equilibrium and showed stable porosity and water retention, which are essential for long-term cell culture and differentiation.

### 3.5. Comparison of Static and Dynamic Culture Conditions

The efficiency of cultured meat production is strongly influenced by the cell culture method. In static cultures, the transport of nutrients, oxygen, and metabolic waste relies on passive diffusion, often resulting in concentration gradients within the scaffold. These gradients can cause uneven cell distribution and reduced proliferation, especially in thick or densely packed scaffolds. In contrast, dynamic culture systems enhance mass transfer through convective flow, improving nutrient delivery and waste removal. This reduces gradients of oxygen and metabolites, thereby enabling more uniform cell growth. Enhanced mass transfer kinetics under dynamic conditions also support higher proliferation rates and facilitate tissue maturation [47,48].

Dynamic culture conditions enhance the oxygen and nutrient supply [49,50], promoting the formation of myoblast bundles closely resembling the muscle tissues and providing a more physiologically relevant environment. However, in random scaffolds, uneven porosity leads to localized deficiencies in nutrient and oxygen exchange, limiting cell viability. In contrast, the structured porous design of the aligned scaffolds promoted cell alignment and positively influenced cell differentiation and muscle tissue formation.

The above-mentioned findings are consistent with the live/dead staining results (Figure 6), further confirming the biocompatibility of both scaffolds. However, the lack of directional structure in the random scaffolds resulted in disorganized tissue formation, making it difficult to mimic the complex architecture of muscle fibers. In contrast, the aligned scaffolds with regularly arranged channels guided the cells to grow along these pathways, leading to a more structured and organized arrangement. The anisotropic structure of the aligned scaffolds supported directional cell growth, resulting in a structure closely resembling that of muscle fibers.

Compared to static culture, dynamic culture provides mechanical stimulation, enhancing nutrient exchange, waste removal, cell attachment, proliferation, and uniform distribution within the scaffold [49,51]. The anisotropic pore structure in the aligned scaffolds facilitated deep cell invasion under dynamic conditions, resulting in uniform distribution along the parallel tubular pores and supporting proliferation. In contrast, the random scaffolds exhibited uneven cell distribution under dynamic culture conditions, possibly due to their irregular porosity and limited structural coherence.

Both scaffolds effectively supported C2C12 cell attachment and proliferation, showing excellent biocompatibility (Figure 6). However, after six days of culture, cell numbers were significantly higher in the aligned scaffolds than in the random scaffolds. The live/dead staining and SEM analyses further revealed that the cells in the random scaffolds grew in a disorganized manner, whereas those in the aligned scaffolds followed parallel channels, effectively mimicking the structure and functions of muscle fibers.

### 3.6. Effect of Scaffold Structure on Cell Differentiation

Compared to the random scaffolds, the aligned scaffolds provided a more favorable environment for C2C12 cell differentiation owing to their oriented porous structure that promoted directional growth and differentiation (Figure 7). The anisotropic structure delivered both mechanical and topological signals, guiding the cells to grow and differentiate in an organized manner along the scaffold. Consequently, the cells formed parallel muscle fiber-like structures that facilitated their maturation into muscle cells.

The random scaffolds, which lacked a clear directional structure, were unable to provide consistent guidance signals to the C2C12 cells. This lack of organization was less effective for differentiation, as muscle fiber formation requires a highly structured and directional environment [10]. Consequently, the cells in the random scaffolds exhibited disordered arrangements, limiting their ability to form mature muscle fibers.

Under dynamic conditions, mechanical stimulation and improved nutrient delivery promoted the cell differentiation of both scaffolds. However, the cells in the aligned scaffolds exhibited organized cytoskeletal arrangements, whereas those in the random scaffolds showed disordered microfilament structures. The anisotropic pore structure of the aligned scaffolds effectively transmitted mechanical and topological cues, leading to the formation of multinucleated cells arranged in parallel, supporting enhanced differentiation.

The random scaffolds lacked a well-defined pore structure, resulting in multidirectional cell differentiation and inconsistent tissue structure. Overall, the oriented porous structure of the aligned scaffolds effectively promoted the directed growth and differentiation of the C2C12 cells, highlighting its potential for muscle tissue engineering [10].

These experimental findings provide insight into how scaffold structure influences muscle cell differentiation. While modeling strategies were not included in the scope of this study, we recognize their importance in understanding scaffold–cell interactions and consider this an important area for future investigation to support the rational design of cultured meat production systems.

### 3.7. Application Potential of Scaffolds

Here, myoblasts proliferated and differentiated along the anisotropic pore structure of the SPI/CA/SA aligned scaffolds, forming muscle fiber-like structures similar to those observed in natural meat tissues. This finding represents a significant step toward the application of scaffolds in cultured meat production. The SPI/CA/SA aligned scaffolds, composed of inexpensive and abundant plant-derived proteins (SPI) and natural polysaccharides (CA and SA), exhibited favorable cytocompatibility, aligning with our goal of sustainable cultured meat development. Overall, the SPI-based scaffolds show great potential for further research and applications.

Cost is a key consideration for the industrial application of cultured meat. The SPI/CA/SA aligned scaffold developed in this study uses plant-derived, inexpensive materials and a simple, energy-efficient fabrication process that does not require costly equipment or toxic reagents. Compared to electrospinning or 3D bioprinting, this method is more suitable for scalable production, supporting its potential as a low-cost scaffold system.

Challenges related to scalability and production efficiency must be overcome to facilitate the large-scale industrial implementation of these scaffolds. Future studies should co-culture multiple cell types on such scaffolds to better replicate the structural complexity of real meat and improve the texture, elasticity, and flavor to enhance their consumer acceptance. With continued optimization and integration into large-scale production [52], SPI/CA/SA aligned scaffolds can facilitate the efficient and cost-effective production of cultured meat.

## 4. Conclusions

In conclusion, we successfully fabricated an aligned cryogel scaffold with mechanical anisotropy using edible materials (SPI, CA, and SA) via directional freezing combined with low-temperature ion crosslinking based on the traditional Japanese *Ito-Kanten* production process. The developed scaffold exhibited a parallel tubular pore structure with excellent mechanical properties, providing an optimal environment for cell adhesion, proliferation, and differentiation. Furthermore, our established scaffold effectively mimics natural muscle tissues, acting as a promising low-cost solution for cultured meat production.

## 5. Materials and Methods

### 5.1. Materials

Soy protein (#61912; protein content: 36.8% *w*/*w*; denaturation temperature: 80 °C) was procured from Daiichi Tanpaku Co., Ltd. (Nagano, Japan). κ-CA (#marugo71) was purchased from Marugo Corporation (Saitama, Japan). SA (#31132-75; viscosity: 1000 cPs) was purchased from Nacalai Tesque, Inc. (Kyoto, Japan) and Fujifilm Wako (Osaka, Japan). Calcein acetoxymethyl ester and propidium iodide were obtained from Dojindo (Kumamoto, Japan). C2C12 myoblasts were obtained from ATCC (CRL-1772).

### 5.2. Preparation of the SPI/CA/SA Cryogel Scaffolds

Hydrogels were prepared by mixing 15% (*w*/*v*) SPI, 3% (*w*/*v*) CA, and 5% (*w*/*v*) SA overnight. SPI, CA, and SA were mixed evenly to form a hydrogel slurry with a volume ratio of 2.5:4:2. The mixture was heated in a hot water bath at 80 °C until it was uniformly dissolved and subsequently cooled to room temperature. This was followed by reheating in the hot water bath at 80 °C to return the mixture to its solution state. The heating and cooling cycles were repeated thrice to obtain a uniform mixture. Subsequently, the well-mixed hydrogel slurry was quickly poured using a syringe into a cylindrical mold with a diameter of 8 mm and height of 8 mm and placed on a copper sheet fixed above liquid nitrogen for the first directional freezing. The frozen hydrogel was immersed in 0.5 M CaCl_2_/KCl solution at 4 °C for low-temperature crosslinking for three days and washed with deionized water for 30 min. The crosslinked hydrogels were placed above liquid nitrogen for secondary directional freezing and freeze-dried to obtain the SPI/CA/SA directional porous scaffolds. The C2C12 cells were seeded onto the SPI/CA/SA aligned scaffolds and cultured for 14 d. SPI/CA/SA random scaffolds were also prepared as controls via freezing in a refrigerator at −30 °C instead of directional freezing, with all the other experimental conditions remaining unchanged (Table 1).

### 5.3. Characterization

#### 5.3.1. Scanning Electron Microscopy (SEM)

The SEM sample preparation for the cell-seeded SPI/CA/SA cryogel scaffolds was conducted following the method reported in reference [35]. Briefly, the scaffolds were rinsed with PBS, fixed with 4% paraformaldehyde at 4 °C for 30 min, and dehydrated through a graded ethanol series (50–100%). Ethanol was then replaced with t-butyl alcohol, and the samples were frozen and lyophilized (ES-2030, Hitachi, Tokyo, Japan). After drying, the scaffolds were sputter-coated with osmium (MSP-1S, Vacuum Device, Ibaraki, Japan) and observed using a desktop SEM (JCM-6000Plus, JEOL, Tokyo, Japan) at 5 kV. Pore area and diameter (Feret diameter) were quantified using the ImageJ software (Ver. 1.54f).

#### 5.3.2. Measurement of Anisotropy

To study the anisotropy of both scaffolds, the samples were cut lengthwise into thin slices, and birefringence was examined under polarized light using a polarized light optical microscope (BX53F2; Olympus, Tokyo, Japan).

#### 5.3.3. Porosity and Swelling Ratio

The porosity and swelling behavior of the SPI/CA/SA aligned and random cryogel scaffolds were evaluated following the established protocols [35]. For porosity, the freeze-dried scaffolds were weighed (*W*_1_), soaked in anhydrous ethanol for 30 min, and reweighed (*W*_2_). Porosity was calculated as follows:

(1) $$Porosity\;(\%)=\left(\frac{W_{2}-W_{1}}{\rho_{E}\cdot V_{H}}\right)\times 100\%$$

where $\rho_{E}$ is the ethanol density (0.79 g/cm^3^) and *V*_H_ is the scaffold volume determined from its dimensions.

For swelling ratio, the dried samples (weight *W*_*D*_) were immersed in PBS at 37 °C and weighed at designated time points (*W*_S_). The swelling ratio (%) was calculated as follows:

(2) $$Swelling\;ratio\;\left(\%\right)=\frac{{W_{S}-W}_{D}}{W_{D}}\times 100\%$$

#### 5.3.4. Mechanical Characterization

Unconfined compression tests were performed at room temperature (25 °C) using a universal testing machine (Force Tester MCT, A&D Company Limited, Tokyo, Japan) with a 500 N load cell and a compression speed of 10 mm/min. The cryogel samples (8 mm × 8 mm × 5 mm) were tested in both longitudinal and cross-sectional directions to evaluate mechanical anisotropy. Stress–strain curves were obtained from the recorded force–displacement data.

### 5.4. Cell Culture

The C2C12 mouse myoblasts were cultured following the method described previously [35] with minor modifications. The freeze-dried aligned and random SPI/CA/SA cryogels (ϕ = 8 mm, h = 8 mm) were sterilized with 70% ethanol, washed with PBS, and preincubated in a culture medium. The cells were seeded at a density of 5 × 10^5^ cells/well and cultured under static conditions for 2 days, followed by dynamic culture on an orbital shaker at 60 rpm. On day 7, the medium was switched to a differentiation medium containing 2% horse serum, and the cells were further cultured for 7 days. For cell recovery, the scaffolds were digested with trypsin, filtered through a 40 µm strainer, and stained with calcein-AM and propidium iodide. The cell number and viability were quantified using a fluorescence microscope (CKX41, Olympus, Tokyo, Japan) and an automated cell counter (CellDrop, DeNovix, Wilmington, DE, USA). The ImageJ software (Ver. 1.54f) was used to calculate the ratio of live to total cells.

### 5.5. Immunofluorescent Staining

After 14 d of differentiation, the aligned and random scaffolds were washed twice with PBS and fixed with 4% paraformaldehyde at 4 °C for 1 h. After washing thrice with PBS, the scaffolds were soaked thrice in 0.05% Tween 20 for 15 min each time, incubated with Blocking One (Nacalai Tesque, Inc., Kyoto, Japan) for 30 min. Then, the samples were stained using AlexaFluor488-Phalloidin (Thermo Fisher Scientific K.K., Tokyo, Japan; 1:200) and Hoechst33342 (Dojindo Laboratories, Kumamoto, Japan; 1:2000) at room temperature for 2 h. Images were acquired using a confocal microscope (Olympus IX-81; Olympus Optical Co., Ltd., Tokyo, Japan).

### 5.6. Statistical Analyses

Data are represented as the mean ± standard deviation (*n* = 3). Tukey’s test was used to compare the differences among groups, with statistical significance indicated as * *p* < 0.05, ** *p* < 0.01, and *** *p* < 0.001.

## Figures and Tables

**Table 1:** Composition of the prepared cryogel scaffold. The table lists the soy protein isolate (SPI), carrageenan (CA), and sodium alginate (SA) contents, along with the type of salt solution used for crosslinking.

Sample	SPI	CA	SA	Salt Solution
**Aligned scaffold**	63%	20%	17%	CaCl_2_/KCl 0.5 M each
**Random scaffold**	63%	20%	17%	CaCl_2_/KCl 0.5 M each

## Data Availability

The original contributions presented in this study are included in the article/Supplementary Materials. Further inquiries can be directed to the corresponding author.

## Supplementary Materials

The following supporting information can be downloaded at https://www.mdpi.com/article/10.3390/gels11040299/s1, Figure S1: Self-gelation states of the hydrogels prepared at 80 °C and cooled to room temperature at different volume ratios. From left to right: Soy protein isolate (SPI)/carrageenan (CA)/sodium alginate (SA) hydrogel solutions with volume ratios of 6:8:4, 5:8:4, 4:8:4, and 3:8:4, respectively; Table S1: Soy protein isolate (SPI), carrageenan (CA), and sodium alginate (SA) concentrations in hydrogel solutions with different volume ratios; Table S2: Compositions of the aligned and random cryogel scaffolds with different SPI/CA/SA ratios; Figure S2: Scanning electron microscopy (SEM) images of the cryogel scaffolds: (A) aligned and (B) random cryogels. The images show the surface, cross-section, and longitudinal section morphologies. Scale bars = 500 µm; Figure S3: Structural and mechanical properties of the aligned and random cryogel scaffolds: (A) Average pore sizes of the aligned scaffolds measured from the SEM images. (B) Average pore sizes of the random scaffolds measured from the SEM images. (C) Compression stress–strain curves of the aligned scaffolds. (D) Compression stress–strain curves of the random scaffolds. * *p* < 0.05, ** *p* < 0.01, and *** *p* < 0.001; Figure S4: C2C12 cell morphology on the aligned scaffolds. Fluorescence microscopy images show cell adhesion and proliferation in the longitudinal sections on days 2, 4, and 6. Green fluorescence indicates the live cells. Scale bars = 100 µm; Figure S5: Compression stress–strain curves of both scaffolds in the cross- and longitudinal sections.

## Abbreviations

The following abbreviations are used in this manuscript:

SPI	soy protein isolate
CA	carrageenan
SA	sodium alginate
SEM	scanning electron microscopy
PBS	Phosphate-buffered saline
FTIR	Fourier-transform infrared
