# Supplementary material for: Development of Biomimetic Edible Scaffolds for Cultured Meat Based on the Traditional Freeze-Drying Method for Ito-Kanten (Japanese Freeze-Dried Agar)
Source: Gels. 2025 Apr 18;11(4):299. doi: 10.3390/gels11040299 (PMC12027230; doi:10.3390/gels11040299)
Supplement: Supplementary file 1 [file gels-11-00299-s001.zip › gels-3587547-supplementary.pdf]

Supplementary Material

Development of Biomimetic Edible Scaffolds for Cultured Meat Based on the Traditional Freeze-Drying Method for *Ito-Kanten* (Japanese Freeze-Dried Agar)

Ping Xia [1], Hiroki Miyajima [1] and Satoshi Fujita [1,2,*]

[1] Department of Frontier Fiber Technology and Sciences, University of Fukui, Fukui,910-8507, Japan
[2] Life Science Innovation Center, University of Fukui, Fukui, 910-8507, Japan
[*] Correspondence: fujitas@u-fukui.ac.jp

Results and Discussion

Optimization of Scaffold Composition

Fabrication of the Soy Protein Isolate (SPI)/Carrageenan(CA)/Sodium Alginate (SA) Aligned and Random Scaffolds

We evaluated the optimal combination of SPI, SA, and CA. As shown in **Table S1**, four SPI/CA/SA mixtures with varying volume ratios (6:8:4, 5:8:4, 4:8:4, and 3:8:4) successfully formed hydrogels at room temperature. The gelated mixtures are shown in **Figure S1**.

Table S1. Soy protein isolate (SPI), carrageenan (CA), and sodium alginate (SA) concentrations in hydrogel solutions with different volume ratios.

SPI:CA:SA	SPI (w/v%)	CA (w/v%)	SA (w/v%)
**6:8:4**	5	1.3	1.11
**5:8:4**	4.4	1.4	1.17
**4:8:4**	3.75	1.5	1.25
**3:8:4**	3	1.6	1.33

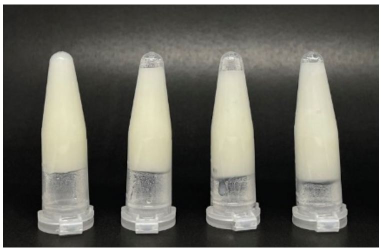

Figure S1. Self-gelation states of the hydrogels prepared at 80 °C and cooled to room temperature at different volume ratios. From left to right: Soy protein isolate (SPI)/carrageenan (CA)/sodium alginate (SA) hydrogel solutions with volume ratios of 6:8:4, 5:8:4, 4:8:4, and 3:8:4, respectively.

The mixtures were subsequently used to fabricate the aligned and random scaffolds. Their final compositions are presented in **Table S2**.

Table S2. Compositions of the aligned and random cryogel scaffolds with different SPI/CA/SA ratios.

SPI:CA:SA	Aligned scaffold	SPI (w/v%)	CA (w/v%)	CA (w/v%)	Random scaffold	SPI (w/v%)	CA (w/v%)	SA (w/v%)
**6:8:4**	**Aligned 1**	67	18	15	**Random 1**	67	18	15
**5:8:4**	**Aligned 2**	63	20	17	**Random 2**	63	20	17
**4:8:4**	**Aligned 3**	58	23	19	**Random 3**	58	23	19
**3:8:4**	**Aligned 4**	51	27	22	**Random 4**	51	27	22

Morphologies of the Aligned and Random Scaffolds

Figure S2A shows that the aligned scaffolds exhibited porous surface structures due to directional freezing, with ice crystals removed via freeze-drying. Longitudinal sections showed aligned channel-like structures. In contrast, random scaffolds exhibited both porous cross- and longitudinal sections, with some pores formed on their surfaces (**Figure S2B**).

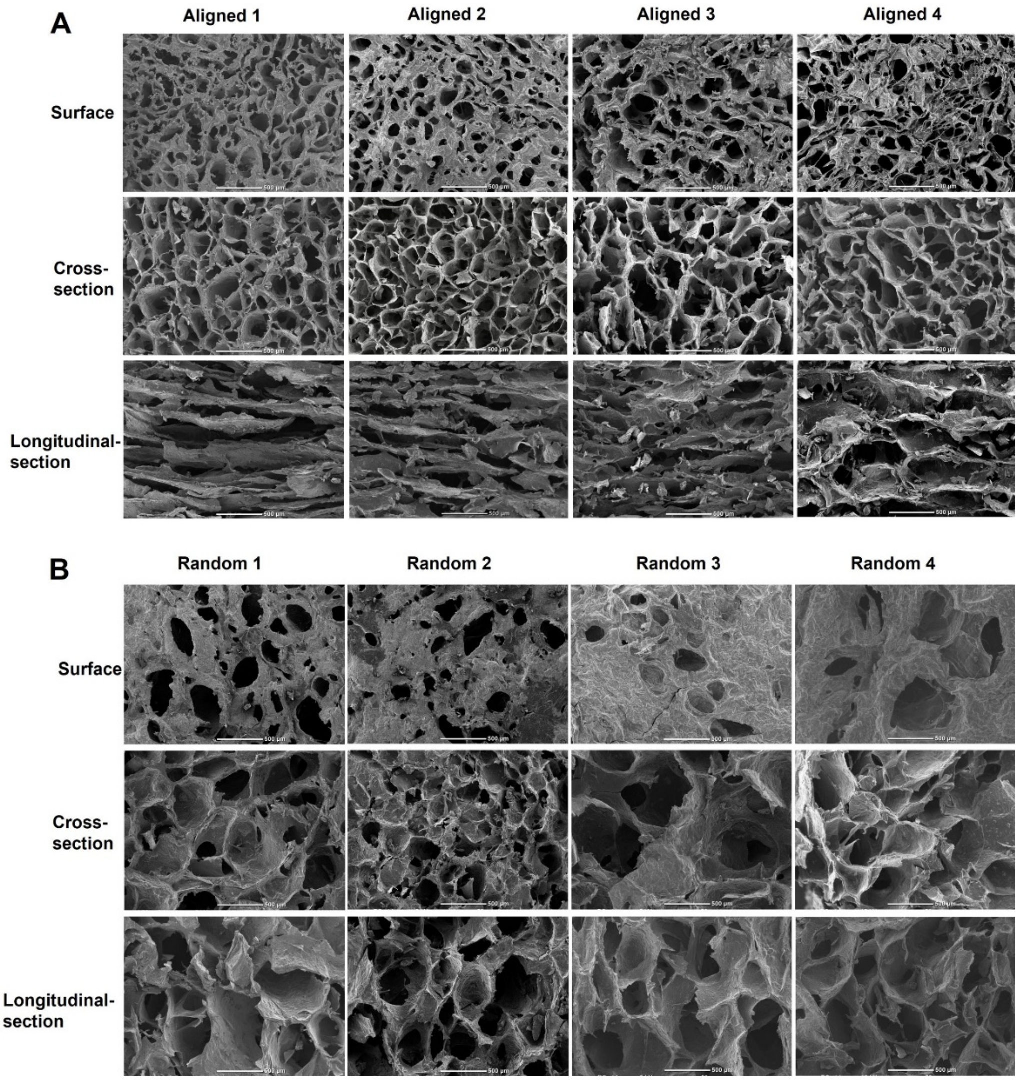

Figure S2. Scanning electron microscopy (SEM) images of the cryogel scaffolds. **(A)** Aligned and (**B)** random cryogels. Images show the surface, cross-section, and longitudinal section morphologies. Scale bars = 500 μm.

Comparison of **Figure S2A** and **B** revealed that the cross-sections of both the aligned 2 and random 2 scaffolds showed relatively dense and uniform pore structures. This uniformity was due to balanced molecular interactions, resulting in a stable and uniform three-dimensional porous network.

Pore Size and Mechanical Properties

As shown in **Figure S3A and B**, aligned 2 scaffold exhibited the smallest pore size among the aligned scaffolds, with an average pore diameter of approximately 150 μm. Similarly, among the random scaffolds, random 2 scaffold exhibited the smallest average pore diameter of approximately 240 μm. Therefore, scaffolds prepared via directional freezing exhibited denser pore structures than those produced using the random method.

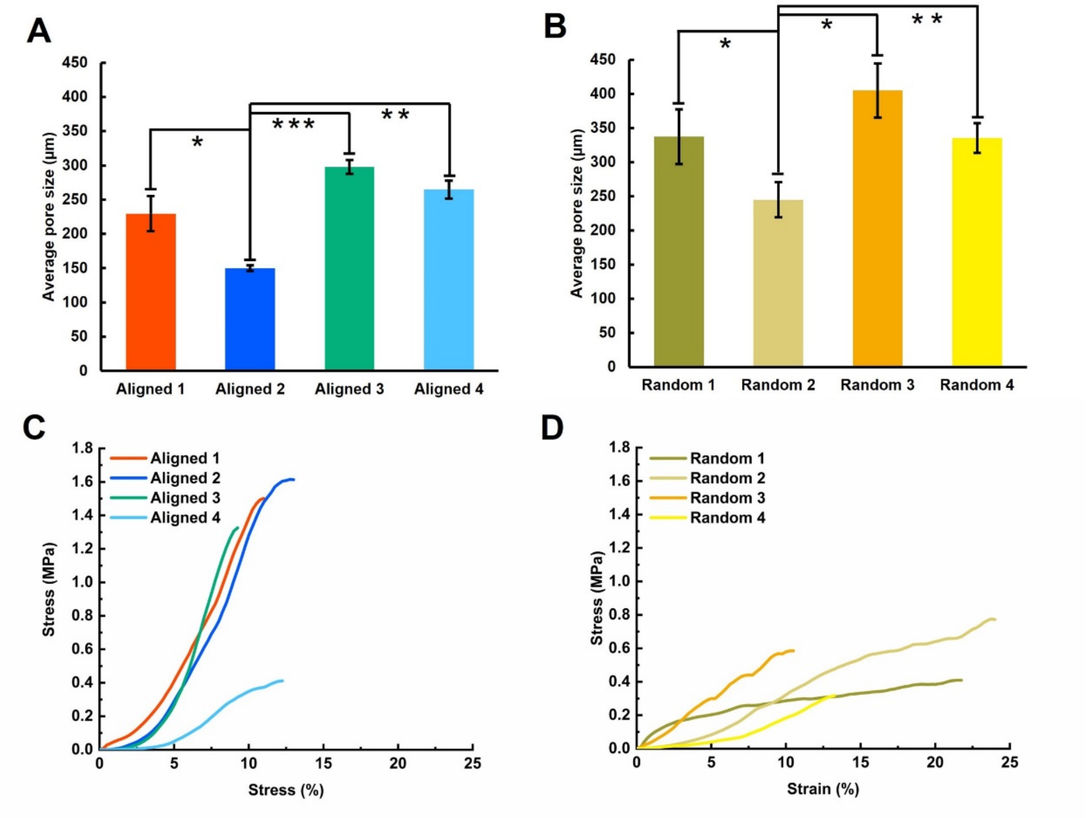

Figure S3. Structural and mechanical properties of the aligned and random cryogel scaffolds. **(A)** Average pore sizes of the aligned scaffolds measured from the SEM images. **(B)** Average pore sizes of the random scaffolds measured from the SEM images. **(C)** Compression stress–strain curves of the aligned scaffolds. **(D)** Compression stress–strain curves of the random scaffolds. *$p < 0.05$, **$p < 0.01$, and ***$p < 0.001$.

As shown in **Figure S3C**, aligned 2 scaffold exhibited the highest compressive strength among the aligned scaffolds, possibly due to its uniform pore architecture. Similarly, random 2 scaffold exhibited the highest compressive strength among the random scaffolds (**Figure S3D**). Overall, both the aligned 2 and random 2 scaffolds formed relatively dense pore structures and exhibited favorable mechanical properties.

Biocompatibility Evaluation of the Aligned Scaffolds

Next, biocompatibilities of the four aligned scaffolds were assessed via live/dead staining (**Figure S4**). Aligned 1 and aligned 2 scaffolds provided favorable cellular environments. On days 4 and 6, the cells exhibited parallel orientations within the scaffold, suggesting strong cell–scaffold interactions. Oriented pore diameters facilitated fibrous cell growth.

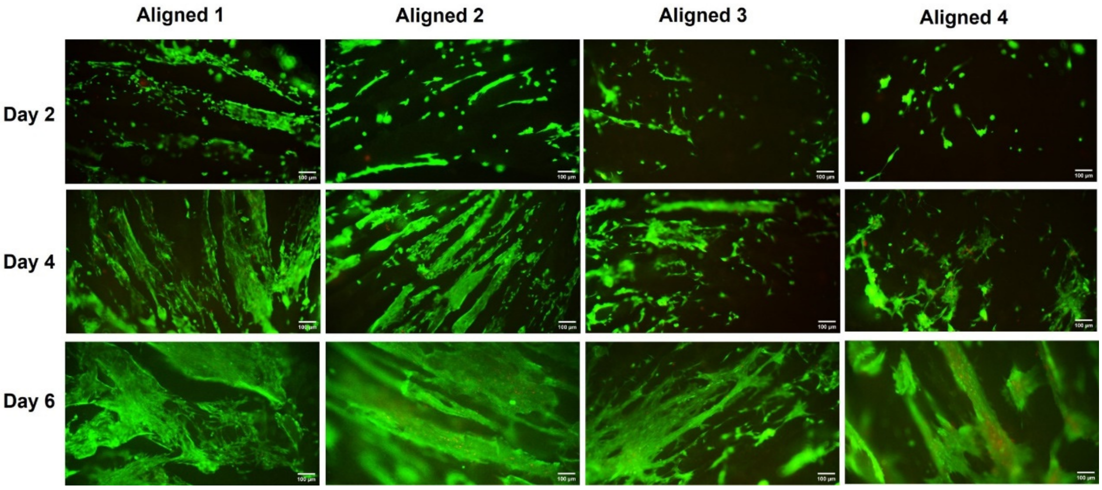

Figure S4. C2C12 cell morphology on the aligned scaffolds. Fluorescence microscopy images show cell adhesion and proliferation in the longitudinal sections on days 2, 4, and 6. Green fluorescence indicates the live cells. Scale bars = 100 μm.

On day 2, aligned 3 and 4 scaffolds showed cell aggregation, suggesting weaker cell–scaffold interactions. This reduced compatibility was due to the low SPI content, which hindered cell attachment and decreased the overall biocompatibility.

Based on these observations, aligned 2 and random 2 scaffolds were selected as the representative scaffolds for further experiments. Aligned 2 scaffold was used as the aligned scaffold for the experimental group, and random 2 scaffold was used as the random scaffold for the control group.

Mechanical Anisotropy

To further investigate the mechanical anisotropy of both scaffolds, cuboid samples were prepared, and compression tests were performed on the longitudinal and cross-sections (**Figure S5**). In the aligned scaffold, compressive strength in the longitudinal section was approximately four times higher than that in the cross-section. This result indicates that the aligned pore structure significantly enhanced the longitudinal compressive strength, thereby conferring mechanical anisotropy.

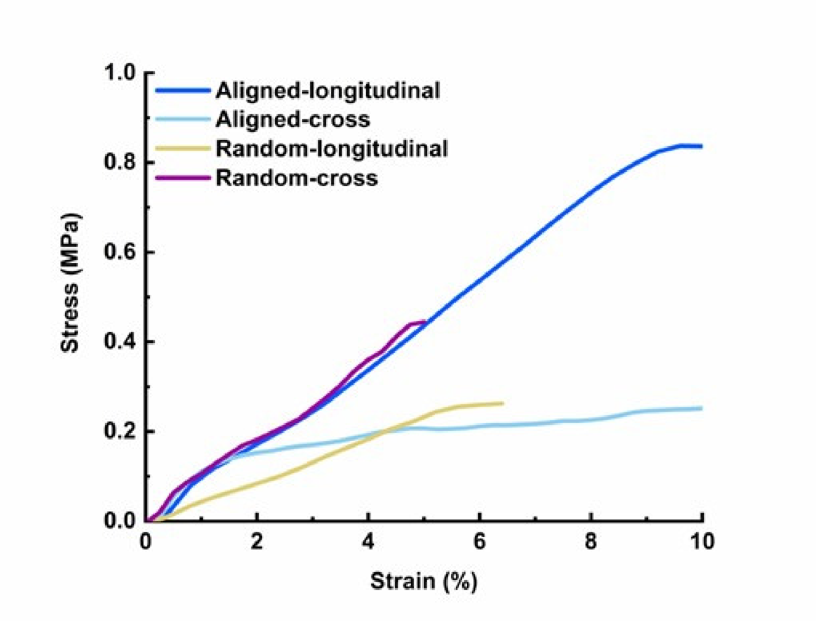

Figure S5. Compression stress–strain curves of both scaffolds in the cross- and longitudinal sections.

Random scaffold exhibited only a small difference in compressive strength between the longitudinal and cross-sectional directions, possibly due to its disordered pore distribution. Moreover, owing to the geometric effects, compressive strength in the cross-section was slightly higher than that in the longitudinal section, further confirming that the random scaffold does not exhibit anisotropic behavior, similar to a previous report [1].

References

1. Hu, T.; Shi, M.; Zhao, X.; Liang, Y.; Bi, L.; Zhang, Z.; Liu, S.; Chen, B.; Duan, X.; Guo, B. Biomimetic 3D Aligned Conductive Tubular Cryogel Scaffolds with Mechanical Anisotropy for 3D Cell Alignment, Differentiation and In Vivo Skeletal Muscle Regeneration. *Chem. Eng. J.* **2022**, *428*, 131017, doi:10.1016/j.cej.2021.131017.
